# Chemical synthetic biology: a mini-review

**DOI:** 10.3389/fmicb.2013.00285

**Published:** 2013-09-23

**Authors:** Cristiano Chiarabelli, Pasquale Stano, Pier Luigi Luisi

**Affiliations:** Science Department, University of Roma TreRome, Italy

**Keywords:** liposomes, minimal cell, protein folding, random sequence, RNA stability, synthetic biology, synthetic cells

## Abstract

Chemical synthetic biology (CSB) is a branch of synthetic biology (SB) oriented toward the synthesis of chemical structures alternative to those present in nature. Whereas SB combines biology and engineering with the aim of synthesizing biological structures or life forms that do not exist in nature – often based on genome manipulation, CSB uses and assembles biological parts, synthetic or not, to create new and alternative structures. A short epistemological note will introduce the theoretical concepts related to these fields, whereas the text will be largely devoted to introduce and comment two main projects of CSB, carried out in our laboratory in the recent years. The “Never Born Biopolymers” project deals with the construction and the screening of RNA and peptide sequences that are not present in nature, whereas the “Minimal Cell” project focuses on the construction of semi-synthetic compartments (usually liposomes) containing the minimal and sufficient number of components to perform the basic function of a biological cell. These two topics are extremely important for both the general understanding of biology in terms of function, organization, and development, and for applied biotechnology.

## INTRODUCTION

Synthetic biology (SB) is one of the most attractive new research areas in biology, and traditionally deals with the bioengineering of new forms of life (generally, but not only, unicellular organisms), which do not exist in nature (Endy, 2005; Andrianantoandro et al., 2006). This is done with the aim of taking the control of their genetic–metabolic network in a predictable way, so that a specific chemical can be produced (for pharmaceutical or biofuel applications), or to use them as biosensors, or for other specific pre-defined purposes. As it happened with the publication of the first self-reproducing organism created by implanting a synthetic genome in a pre-existing cell (Gibson et al., 2010), ethical issues are often related to the bioengineering of natural living systems, especially as soon as the technical progresses allow the manipulation of ever-complex organisms.

On the other hand, there is a second “soul” of SB. The second soul, as we have defined in other articles (Luisi, 2007; Chiarabelli et al., 2009, 2012), and in a book (Luisi and Chiarabelli, 2011), has to do with the idea of synthesizing in the lab biological structures which do not exist in nature, and without the use of gene manipulation – but rather with the tools of simple chemistry manipulation. We have dubbed this second line of thought as “chemical synthetic biology” (CSB). CSB is concerned with the synthesis of chemical structures such as proteins, nucleic acids, vesicular forms, and others which do not exist in nature. This is done for two reasons: first, CSB approaches allow understanding the roots of biological function and organization, because it allows to test hypotheses about the reasons according to which biology works. Second, as for SB, it might have a tremendous impact on biotechnology, generating an entire set of tools for nanomedicine, diagnostic, drug/gene delivery, bioengineering, biosensoring, etc., based on artificial molecules. Examples of CSB approaches span from searching for nucleic acid alternatives, such as furanose-based DNA (Bolli et al., 1997), peptide-nucleic acids (PNAs; Egholm et al., 1993) and their conjugates (Alajlouni and Seleem, 2013), to the novel work on synthetic genetic polymers (XNA) capable of heredity and evolution (Pinheiro et al., 2012), and to synthetic genetic codes (Wong and Xue, 2011); and, on the other hand, to proteins composed by random amino acid sequences (Chiarabelli et al., 2006a, b), or from a subset of amino acids (Doi et al., 2005), or other unnatural building blocks, or by combinatorial approaches (Urvoas et al., 2012). In addition to these examples of CSB of “parts,” there is a flourishing research on CSB of “systems,” and in particular that one focused on the construction of synthetic cells (Luisi et al., 2006b), whose achievement represents the most ambitious goal of CSB.

In this mini-review we will focus on two CSB projects that we are currently developing, namely, the “Never Born RNAs (NBRNA)/proteins” [Never Born Biopolymers (NBB)] and the “Minimal Cell.” Due to space limitation we cannot present several interesting and original reports on related subjects, and we simply recapitulate our main results and the comments on them, which have been published in previous reviews (Luisi et al., 2006b; Chiarabelli et al., 2009, 2012), with the addition of some most recent advancements, when available. The NBB project is related to the question why and how the existing protein or RNA structures have been selected out, with the underlying question whether they have something very particular from the structural or thermodynamic point of view (for example, the folding). The Minimal Cell project is focused on the construction of cell models having the minimal and sufficient number of components to be defined as living. For this purpose, liposomes are used as shell membranes, and attempts are made to introduce in the interior a minimal genome. The current state-of-the-art in this research is the synthesis of functional protein inside liposomes, and the study of the corresponding artificial cell reactivity. Before presenting in more details these two projects, however, a short paragraph on general epistemic considerations on SB and CSB is presented.

## SHORT EPISTEMIC REMARKS

Theoretical thinking is too often neglected in modern biotechnological education programs, and contemporary researches seldom consider their studies from the epistemic viewpoint. Quite specific epistemic features characterize SB and CSB approaches. Classical SB, funded on the concept of bioengineering, is a *teleological* enterprise. In fact, as when an engineer plans an aircraft, a synthetic biologist plans (and later builds) a genetically modified organism for a specific purpose. This is generally done by removing, adding, or exchanging biological parts of an organism. Although *reductionism* can be guessed as a guiding principle for such operation, actually, in order to work, the new modules must be *integrated* with the other pre-existing biochemical networks, so that a *systemic* thinking is required. Remarkably, whereas teleology dominates SB, *teleonomy* features natural biological evolution.

On the other hand, CSB often faces dichotomies as *contingency* and *determinism*. For example, the study of random proteins for determining whether natural proteins are the only possible function-bearing structures, answer the question whether life – as we know it – has followed a deterministic path or it is rather a product of contingent, punctual, historical unrepeatable events. The philosophical implication of constructing synthetic living cells from non-living parts deals instead with the concepts of *emergent* properties – those properties that cannot be reduced to the properties of the parts composing the system. In addition, this issue directly brings about the problems connected with the deep understanding of “what is life,” its definition, and its recognition. In this respect, the Maturana–Varela concepts of *autopoiesis* and *cognition* (Varela et al., 1974) constitute a powerful and elegant theoretical framework. More detailed epistemological considerations can be found in Luisi (2011).

## NEVER BORN BIOPOLYMERS

The idea on the NBB moves from the observation that the extant collection of proteins and RNA molecules, currently present in living organisms, are only a minor part of the all theoretical possible sequences (de Duve, 2002; Luisi, 2003; Xia and Levitt, 2004; Chiarabelli et al., 2006a,b, 2011; Dryden et al., 2008; LaBean et al., 2011). For example, the number of possible different peptides of 50 amino acid residues synthesized using the 20 natural amino acids is equal to 20^50^, i.e., 10^65^. The difference between the number of possible proteins and the number of those actually present in living organisms is comparable to the difference existing between a grain of sand and the entire Sahara desert (Luisi, 2006). The number of possible different proteins becomes even greater if we take into account the living organisms, where the average length of proteins is much greater (Lipman et al., 2002). In the same way these considerations can be broadened to the RNAs.

On the basis of this observation, the question whether a functionality is a common feature or a rare result of natural selection is of the utmost importance to elucidate the role of biopolymers in the origin of Life and to fully exploit its biological potential. As an obvious consequence, it has been decided to explore the protein and RNA sequence spaces in search of random molecules presenting a stable fold, by assays set up in our laboratory (Chiarabelli et al., 2006b; De Lucrezia et al., 2006b; Anella et al., 2011).

**FIGURE 1:**
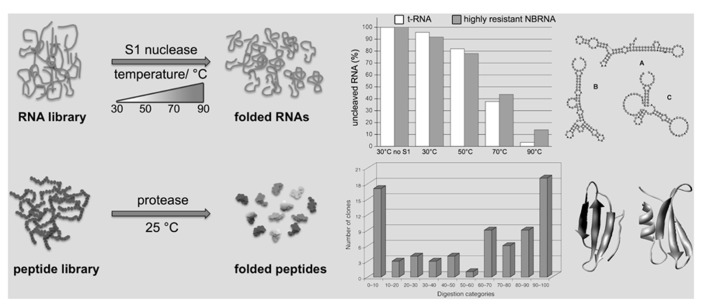
**Schematic representation of the random RNAs and peptides selection procedures.** An RNA library, containing folded and unfolded (or not well-folded) sequences, is treated with S1 nuclease at various temperatures. In the corresponding graphic the residual amount (%) of a tRNA as control (white bars) in comparison to a highly resistant random RNA (NBRNA; gray bars) at different temperatures. The corresponding secondary structure predictions for the NBRNA at different temperatures are shown (A: 24 ≤ *T* ≤ 63°C; B: 64.5 ≤ *T* ≤ 72°C; C: 74 ≤ *T* ≤ 91°C). Only folded RNA sequences are preserved, whereas the other are digested. Similarly, a peptide library, containing folded and unfolded (or not well-folded) sequences, is treated with a protease. Only folded peptide sequences are recovered, whereas the unfolded one are digested. In the graphic [reproduced from Chiarabelli et al. (2006b), with the permission of Wiley] the tested peptides are divided in categories according to the different levels of resistance against enzymatic digestion shown by each peptide sequence; for example, class 0–10 displays a digestion between 0 and 10%. Two tertiary structure predictions examples of folded resistant NBP are shown.

In order to be proteins, amino acid chains have to be folded; the Never Born Proteins (NBP) project is directed toward the discrimination between folded and unfolded chains in a random ensemble of synthetic polypeptides. The approach involves a “total randomization” with no bias toward any given structural or functional property, leading almost necessarily to novel proteins not present in nature, the NBP (Chiarabelli et al., 2006b; Luisi et al., 2006a). The experimental procedure developed to pursue this goal is based on the concept that folded polypeptides are more protected against digestion by a protease than unfolded ones. The first attempts to combine a library-production method with the selection of folded protein by proteolysis belong to Kristensen and Winter’s (1998). In that case the starting point has been the selection of variants with improved properties from known extant proteins. The originality of our procedure, with respect to literature, lies in the library itself, being made by *de novo* completely random sequences not present in nature and in the insertion of a specific short fixed sequence within a total random polypeptide sequence to create the library. Such short sequence is specifically recognized, so that folded peptides have less chances to be enzymatically digested than unfolded ones. The NBP selection method links the proteolytic resistance with the selective recovery, useful to get new folded peptides (**Figure 1**). With our approach, a library of 50 residues long random peptides has been produced and screened (**Figure 1**, bottom), and the work demonstrates that the selection of folded random proteins is feasible (Chiarabelli et al., 2006b, 2009, 2012; Luisi and Chiarabelli, 2011). Preliminary circular dichroism studies further support our conclusions based on limited digestion. Furthermore, because the NBP Project born on the basic idea to test whether the extant proteins have no extraordinary physical properties at all, it is reasonable to say that the natural proteins could have been selected by chance among an enormous number of possibilities of quite similar compounds. They came out by “chance,” and it happened that they were capable of fostering cellular life.

Confirming the idea that protein fold is a general feature of relatively long polypeptide chains there are a lot of additional studies. Ribosomal display, which allow the screening, selection, and evolution of functional proteins, was used by Hanes and Plückthun (1997) to investigate antibody sequences variations, by maintaining however the full antigene-binding capacity. Recent directions in ribosome display technologies are illustrated by Plückthun (2012). Yomo and colleagues (Yamauchi et al., 2002) investigated the possibility of selecting an active enzyme by *in vitro* evolution starting from the very limited number of 10 random sequences, showing that a significant fraction of all possible sequences may have functions, at least binding activity, using a simple selection method applied to relatively short random proteins (about 140 amino acids). In a second experiment, starting from the same library as before, they also demonstrated the evolvability of both a single soluble arbitrary sequence toward a structure with a biological function, for example molecular folding and recognition (Hayashi et al., 2003), and a insoluble random polypeptide toward a soluble one (Ito et al., 2003). Importantly, a well-structured molecular fold is a pre-requisite for more interesting catalytic functions.

Even the idea to use a binary pattern of polar/non-polar amino acid scheme periodicity (Kamtekar et al., 1993; Wei et al., 2003; Hecht et al., 2004) or a subset of amino acids (glutamine, leucine, and arginine; Davidson et al., 1995) to construct *de novo* libraries with stable three-dimensional structures demonstrate that the frequency of folding in random libraries is higher than expected, and that it is possible to select proteins with desired characteristics. Unfortunately, in the last years we do not have more examples on the study of totally random proteins with respect to folding and structure analysis.

Novel reports have highlighted the role of combinatorial design to produce and select 102 residues long proteins with capability of sustaining *E. coli* growth (Fisher et al., 2011), as well as the importance of *in silico* approaches for the exploration of the sequence space (Prymula et al., 2009; Cossio et al., 2010; De Lucrezia et al., 2012).

In a similar way as described for the NBP has been possible to investigate the random RNA (NBRNA) characteristics. Knowing the firm relation between structure and function of biological molecules we decided to precede the random RNAs functional exploration with some structural studies using the RNA Foster (RNA folding stability test). The assay allows to determine the presence and thermal stability of secondary domains in RNA molecules by coupling enzymatic digestion with temperature denaturation (**Figure 1**, top). The Foster is capable to quantitatively determine the fraction of folded RNAs in function of temperature (De Lucrezia et al., 2006a). It employs a specific nuclease (S1; Vogt, 1973) to cleave at different temperatures single-strand RNA sequences, monitoring the presence of double-stranded domains and indirectly any possible structure. In fact, folded RNAs are more resistant to S1 nuclease than unfolded ones, namely the latter are degraded faster than the former. In addition, we exploited the capability of nuclease S1 to work over a broad range of temperatures to probe RNA secondary domain stability at different conditions. In fact, an increase in temperature destabilizes the RNA fold, inducing either global or local unfolding. Consequently, the RNA becomes susceptible to nuclease attack and is readily degraded (De Lucrezia et al., 2006b; Anella et al., 2011). In few words the most stable sequences at high temperature will be those with a more stable secondary and possibly tertiary structure.

So far, the most general result of our studies lies in the demonstration that RNAs have the capacity to fold into compact secondary structures, even in absence of selective pressure (Anella et al., 2011). This confirm our hypothesis that molecules involved in nowadays life do not have exclusive features as far as the ability to adopt a stable fold is concerned.

Unexpectedly, one of the sequence analyzed has a stability even higher than a tRNA, used as control, at 70°C, with an approximately melting temperature higher than 80°C. These results are absolutely outstanding and can be used to provide directions and suggestions for further studies concerning the functional properties of RNAs, in the early evolution scenario as well. In our laboratory we are trying to study more in detail the structure characteristic of NBRNA using more complex libraries and secondary and tertiary structure predictions as well as spectroscopic methods.

## SEMI-SYNTHETIC MINIMAL CELLS

When we look at modern living cells it is difficult not to remain astonished by the beautiful complexity of thousands of intricate genetic–metabolic complexes occurring in the tiny cellular environment, and we ask how this complexity was originated by spontaneous generation and later shaped by the evolution. However, there was a time when cells were much simpler that modern one, and still being “alive,” i.e., capable of self-maintenance, self-reproduction, and with the capacity of evolve. How can we study such simple and primitive cells? These biological entities do not exist any more in nature, and therefore the synthetic (constructive) approach – typical of CSB – is the only way to get insights into the physical and chemical constraints that ruled the emergence of early cells in ancient times. However, any attempt to construct a complex system such as a living – yet primitive – cell needs the knowledge of the minimal biological organization that characterizes life. In this context, the theory of autopoiesis, introduced in the 1970s by Humberto Maturana and Francisco Varela (Varela et al., 1974), is of great help. Autopoiesis (self-production) says that a living cell is a physical object (composed by reactive molecules), which: (1) distinguishes itself from the environment thank to a well-defined semi-permeable boundary, (2) encloses networks of reactions that transform the precursors available in the environment in the same molecules that form the reaction network, (3) despite the continuous turnover of its molecular constituents, the autopoietic system maintain its own identity in terms of (dynamic and spatial) organization. Thanks to these theoretical guidelines, can we build minimal autopoietic cells in the laboratory? The research project called “Minimal Cell” aims at answering this question in several ways. One possibility is the construction of minimal cells basing on allegedly primitive molecules, such as fatty acids, ribozymes, simple primitive peptides. A simple ribozyme-in-liposome model has been proposed (Szostak et al., 2001), but this route, although fascinating, is hampered by the very limited reactivity displayed by known ribozymes, and by the lack of viable catalytic short peptides (despite some notable cases, such as the Ser-His catalysis, see Gorlero et al., 2009; Wieczorek et al., 2012; Adamala and Szostak, 2013), and notwithstanding the instead rather rich and well-characterized properties of fatty acid vesicles (Walde et al., 1994; Berclaz et al., 2001; Stano et al., 2006; Zhu and Szostak, 2009). Another possibility deals with totally synthetic systems, based for example on polymers, PNAs, elaborated transition metal catalysts, etc. (Rasmussen et al., 2004), aiming at creating a totally synthetic minimal cell. Water-in-oil emulsion droplets, where compartmentalized reactions can be carried out, as pioneered by Tawfik and Griffiths (1998), are also alternative synthetic systems to build cellular models, but they lack of the semi-permeable lipid bilayer that characterizes biological cells.

**FIGURE 2:**
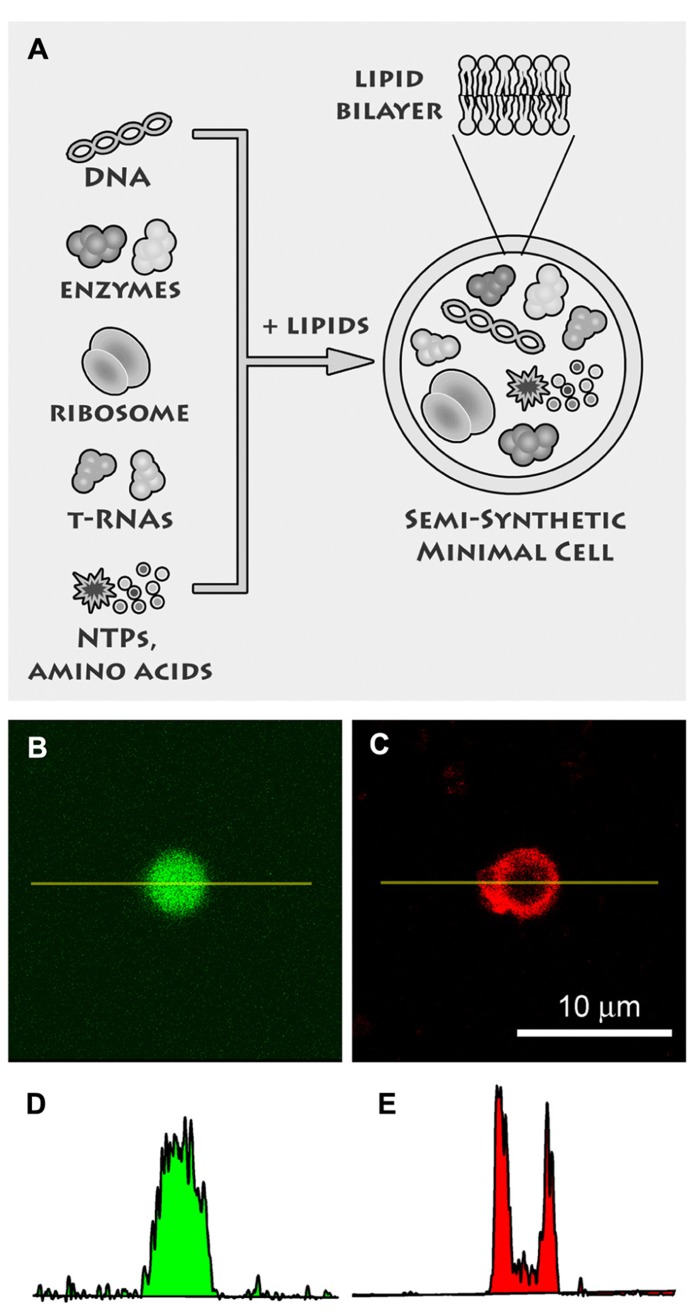
**Semi-synthetic minimal cells.** **(A)** Semi-synthetic minimal cells are based on the encapsulation of the minimal number of biological molecules, like DNA, ribosomes, tRNAs, enzymes, amino acids, nucleotides, etc., inside lipid vesicles. Currently it is possible to encapsulate the whole transcription–translation machinery so that proteins are synthesized inside the synthetic cell. The goal of research is the self-reproduction of the minimal cells thanks to the simultaneous (and possibly coupled) production of internal and membrane components. Reproduced from Chiarabelli et al. (2009) with the permission of Elsevier. **(B)** Confocal fluorescence image of a liposome, prepared by the droplet transfer method, which contains the whole transcription–translation machinery (*E. coli* cell extracts) for producing a protein from the corresponding DNA sequence. In this case, the enhanced green fluorescence protein (eGFP) was synthesized, as evidenced by the green fluorescence. **(C)** The liposome membrane were made visible after Trypan blue addition, which binds to phospholipid bilayers and becomes red fluorescent upon 543 nm excitation. **(D,E)** Quantitation of fluorescence along the yellow lines in **(B,C)** gives typical bell-shaped and U-shaped profiles, respectively, for water-soluble (eGFP) and membrane-bound (Trypan blue) fluorochromes.

We believe that a third way is actually the most fruitful one, because it has been successfully used to shed light on the emergence primitive cells, and at the same time it promises remarkable applications in biotechnology (Jewett and Forster, 2010), nanomedicine (Leduc et al., 2007), drug delivery, and in modern bio-chem based information/communication technologies (ICTs; Stano et al., 2012). This is the “semi-synthetic” approach (Luisi et al., 2006b; Stano et al., 2011), consisting in the construction of cell-like structures by encapsulating the minimal number of available DNA, RNA, proteins inside liposomes (**Figure 2**). Currently this approach stems from the convergence of liposome technology and cell-free technology and it is investigated by several groups, as witnessed by the richness of the recently published original work (Budin et al., 2012; Hamada and Yoshikawa, 2012; Maeda et al., 2012; Kobori et al., 2013; Lentini et al., 2013; Nourian and Danelon, 2013). Several important concepts and technical issues play essential roles in semi-synthetic minimal cells, such as (1) life as an emergent property, (2) the minimal genome, (3) the production of functional water-soluble and membrane proteins, (4) the conditions for core-and-shell self-reproduction, (5) “spontaneous” versus “directed” preparation methods, and many others.

For example, several studies revealed that the essential housekeeping functions of living cells are encoded in a minimal number of genes, around 200 (Gil et al., 2004), as derived from comparative genomic analysis carried out on the smallest living organisms (often intracellular parasites or symbionts). About one half of the minimal genome is devoted to the specification of proteins and RNAs dedicated to the protein synthesis, without doubts the most important process, together the nucleic acid replication and the lipid synthesis (but consider that the latter two processes are only possible thank to enzymes). It is therefore not quite surprising that the current state-of-the-art in minimal cell research focuses on protein synthesis inside liposomes. Several advancements have been reported from the time of the pioneer report from Oberholzer et al. (1999). In particular, proteins like the green fluorescent protein, β-galactosidase, Qβ-replicase, α-hemolysin, and T7 RNA polymerase have been successfully synthesized inside liposomes, according to a variety of experimental design, aimed at functionalizing synthetic cells with pores, RNA-replication, two-steps genetic cascades, etc. (for a review, see Stano et al., 2011). Most of the more recent studies are carried out by incorporating – inside liposomes – of the minimal number (ca. 80) of transcription/translation macromolecules, i.e., the PURE system (Shimizu et al., 2001). The issue of lipid production has been faced by reconstituting the “lipid-salvage-pathway” inside liposomes, a four-steps mini-metabolic route that converts glycerol-3-phosphate into phosphatidylcholine (Schmidli et al., 1991; Kuruma et al., 2009). The low yield associated to the involvement of the membrane enzymes that catalyze these transformations has prevented efficient lipid production, so that the direct observation of a spontaneous growth and division due to intraliposome lipid production is still missing.

There are, however, novel and intriguing advancements in exploring the emergence of minimal cells from separated components. In fact, we recently investigated in details the physics of solute encapsulation inside liposomes spontaneously formed by lipid self-assembly – a key event for the emergence of primitive cells. Driven by our intriguing observations on the success of solute encapsulation and protein synthesis inside 200 nm (diameter) vesicles (Souza et al., 2009), we asked what are the mechanisms that allow the capture of very large number of solutes inside a closed lipid compartment. By using ferritin, ribosomes, and ribo-peptidic complexes, we revealed the true intraliposome solute distribution by direct counting the number of entrapped macromolecules via cryo-transmission electron microscopy (Luisi et al., 2010; Souza et al., 2011, 2012). Interestingly, although the majority of liposomes were “empty” or contain the expected number of solutes, we found about 0.1% of liposomes containing a very high number of solutes, exceeding by at least one order of magnitude the expected concentration. In other words, liposomes can spontaneously concentrate macromolecular solutes in their cavity, providing a mechanism for the emergence of cellular metabolism even starting from a diluted solution. In fact, this mechanism might provide a rational explanation on the onset of intraliposome complex reactions that cannot occur in bulk phase due to extreme dilution. We have recently assayed such realistic scenario by using the synthesis of green fluorescent protein synthesis as a metabolic model (Stano et al., 2013).

## CONCLUDING REMARKS

An additional dimension has been added to SB, namely the CSB approach, based on the concept of producing biological structures alternative to the natural ones, by using chemical and biochemical technology.

In this mini-review we have shortly commented two of our projects in CSB, namely the “Never Born Biopolymers” and the “Minimal Cells” projects. The corresponding investigations are based, respectively, on the exploration of peptide- and RNA-libraries, and on the encapsulation of solutes inside lipid vesicles. These approaches give a rather rich variety of novel forms and corresponding novel ideas, which may be relevant for understanding how biological systems are constructed and works, as well as for potentially new biotechnological applications. For example, NBRNAs might provide insights into the so-called RNA world hypothesis, but also provide sequences with unexpected biological effects. NBRNAs might become novel therapeutical agents. Minimal cells, when properly designed, might find applications in advanced drug delivery approaches, and would not only serve as model or primitive cells. CSB is a large field in which basic science and applicative research combine together, and we are confident that it will bring about significant advancements.

## Conflict of Interest Statement

The authors declare that the research was conducted in the absence of any commercial or financial relationships that could be construed as a potential conflict of interest.
